# Mother–infant social and language interactions at 3 months are associated with infants’ productive language development in the third year of life

**DOI:** 10.1016/j.infbeh.2024.101929

**Published:** 2024-04-06

**Authors:** Yaara Endevelt-Shapira, Alexis N. Bosseler, Julia C. Mizrahi, Andrew N. Meltzoff, Patricia K. Kuhl

**Affiliations:** a Institute for Learning & Brain Sciences, University of Washington, USA; b Department of Psychology, University of Washington, Seattle, WA, USA; c Department of Speech and Hearing Sciences, University of Washington, USA

**Keywords:** Maternal sensitivity, Language development, Infant directed speech, Conversational turns, Social interaction

## Abstract

Previous studies underscore the importance of social interactions for child language development—particularly interactions characterized by maternal sensitivity, infant-directed speech (IDS), and conversational turn-taking (CT) in one-on-one contexts. Although infants engage in such interactions from the third month after birth, the prospective link between speech input and maternal sensitivity in the first half year of life and later language development has been understudied. We hypothesized that social interactions embodying maternal sensitivity, IDS and CTs in the first 3 months of life, are significantly associated with later language development and tested this using a longitudinal design. Using a sample of 40 3-month-old infants, we assessed maternal sensitivity during a structured mother–infant one-on-one (1:1) interaction based on a well-validated scoring system (the Coding Interactive Behavior system). Language input (IDS, CT) was assessed during naturally occurring interactions at home using the Language ENvironment Analysis (LENA) system. Language outcome measures were obtained from 18 to 30 months of age using the MacArthur-Bates Communicative Development Inventory. Three novel findings emerged. First, maternal sensitivity at 3 months was significantly associated with infants’ productive language scores at 18, 21, 24, 27, and 30 months of age. Second, LENA-recorded IDS during mother–infant 1:1 interaction in the home environment at 3 months of age was positively correlated with productive language scores at 24, 27, and 30 months of age. Third, mother–infant CTs during 1:1 interaction was significantly associated with infants’ productive language scores at 27 and 30 months of age. We propose that infants’ social attention to speech during this early period—enhanced by sensitive maternal one-on-one interactions and IDS—are potent factors in advancing language development.

## 1. Introduction

Research suggests that early social interaction plays a significant role in language development during infancy (Brooks & Meltzoff, 2008; Lytle & Kuhl, 2017; Rowe & Snow, 2020; Rowe & Weisleder, 2020; Tamis-LeMonda et al., 2014; Tomasello, 2019). From birth, infants experience social contingencies with their caregivers and other adults, and research indicates that both the quality and quantity of early social interactions are linked to a child’s brain growth and behavioral advancements. The quality of these social interactions—including the degree of parental sensitivity (Madigan et al., 2019; Tamis-LeMonda et al., 2014), as well as parental speech style and social context of the interaction (Ramírez-Esparza et al., 2014, 2017a, 2017b)—have been shown to be significantly associated with early language learning.

An important milestone in language acquisition is phonetic learning. Infants begin life perceiving phonetic differences used to distinguish words across all languages, but between 6 and 12 months of age infants show a perceptual narrowing in their speech perception abilities that is specific to their native language (Best & McRoberts, 2003; Kuhl et al., 2006; Werker & Tees,1984). Experimental evidence shows that within this window, social interaction with a live person is necessary to learn new phonetic units and words (Kuhl et al., 2003; Conboy & Kuhl, 2011)—infants who were exposed to a new language during live social interaction learned to discriminate the sounds of that language, whereas infants who experienced the same language via TV or audio only did not (Kuhl et al., 2003; Conboy & Kuhl, 2011). There is evidence that infants’ social behaviors during these language learning episodes, evidenced by infant eye gaze and speech-like vocalizations, were significantly associated with the degree to which infants learn both new sounds and words (Conboy et al., 2015; Lytle et al., 2018). These data suggest that social interaction may be essential to early language learning (Kuhl, 2007).

Social interaction and language input provide opportunities for language learning, including conversational turns between the infant and caregiver and adaptations in the caregiver’s speech style, such as infant-directed speech, that are directly related to the sensitivity of the social partner. Infants are exposed to social information from birth and by 3 months of age have begun to attune to the social environment, for example, by engaging in reciprocal exchanges during face-to-face social interactions with their caregivers (Beebe et al., 2016; Lavelli & Fogel, 2002), participating in conversation-like turn-taking (Gratier et al., 2015), and changing their visual, facial, and vocal behaviors in response to their caregiver’s social cues (Feldman, 2007b; Meltzoff & Moore, 1998). Despite the emergence of these social abilities early in development, there are scant data about the specific link between infants’ social environment during this early window and subsequent language development.

In the current study, we examine whether parent-infant interactions at 3 months of age are associated with future language development. Previous research points to strong links between maternal sensitivity, IDS, and conversational turns (CT), and language outcomes. We hypothesize that these aspects of social interactivity can be measured very early in development, prior to the well-documented periods for phonetic and word learning, and that social interactivity scaffolds language growth by enhancing infants’ interest in and attention to social stimuli, which in turn, fosters learning. In the present study we measure specific early mother–infant social interaction attributes, including maternal sensitivity and speech input to the infant at 3 months of age, and their relation to subsequent language growth.

During the first months of life, mothers naturally “synchronize” their behaviors with infants’ moments of alertness (Feldman, 2007a; Fleming et al., 1999). For example, mothers typically adapt their speech according to the infants’ non-distress vocalizations (Hsu & Fogel, 2003). Moment-by-moment adaptation to infants’ cues is an important aspect of sensitive caregiving. Parental sensitivity has long been recognized as a determinant of secure attachment relationships (Bakermans-Kranenburg et al., 2003; De Wolff & van Ijzendoorn, 1997) and has been reported to have a role in cognitive and social development (Belsky & Pasco Fearon, 2002; Feldman et al., 2004; Madigan et al., 2019). The importance of parental sensitivity during infants’ prelinguistic period for language development and communication has been previously highlighted (Madigan et al., 2019; Tamis-LeMonda et al., 2014). Studies have demonstrated that caregivers’ verbal response to infants’ babbling provides real-time effects on the development of prelinguistic vocalizations and communication skills. In one study, mothers of 9.5-month-old infants were instructed to provide models of vocal production timed to be either contingent or noncontingent on their infants’ babbling. In the contingent condition mothers were instructed to verbally respond to their infant’s babbling, while in the noncontingent condition mothers’ verbal input was temporally dissociated from their infants’ babbling. Infants in the contingent condition modified their babbling in accordance with the phonological structure present in their mothers’ contingent utterances, whereas infants in the noncontingent condition did not (Goldstein & Schwade, 2008).

In addition to studies showing that infants make real-time modifications in their babbled utterances following contingent verbal feedback, several longitudinal studies have examined the long-term effects of maternal sensitivity and responsiveness to infants’ cues on language development. It has been shown that maternal verbal sensitivity at 9 months of age is predictive of 13-month child language comprehension (Baumwell et al., 1997). It was further shown that maternal affective matching responses to their 9-month-old infants’ emotional displays predicted infants’ language achievements at 21 months of age (Nicely et al., 1999).

Other studies examined longitudinal interrelations between preverbal mother–infant interaction at 6 months of age and children’s vocabulary development and found that maternal responsiveness during interactive play correlates positively with vocabulary size at 24 months of age (Olson et al., 1986). It was further shown that maternal responsiveness predicts not only vocabulary size, but also the timing of language milestones. Maternal responsiveness at both 9 and 13 months of age predicted children’s timing of achieving language milestones including first linguistic imitations, first words, 50 words in expressive language, combinatorial speech, and the use of language to talk about the past (Tamis-LeMonda et al., 2001). Another study found that maternal sensitivity and maternal language at 5 months each uniquely predicts child language at 49 months (Bornstein et al., 2020).

Interestingly, maternal sensitivity measured in different developmental stages appears to relate to language development at different stages. A study measuring the effects of maternal sensitivity from ages 6 to 36 months on children’s language development at 15, 24, and 36 months of age found that sensitive maternal interaction positively affected children’s later expressive language in the second and third years of life. However, early maternal sensitivity at 6 months of age did not show a substantive correlation with language skills until 36 months (Leigh et al., 2011).

It has also been shown that maternal responsiveness during the first year of life (measured when infants were 9 and 12 months) is a stronger predictor of infant vocabulary size at 18 months than maternal depression or anxiety scores (Brookman et al., 2023). Importantly, the significant contribution of maternal sensitivity for language development is evident not only in typically developing infants, but also in children with language skills that develop more slowly (la Paro et al., 2004; Levickis et al., 2014) and in children with developmental disorders such as Fragile X syndrome (Warren et al., 2010). These findings underscore the potential importance of social interactions and maternal sensitivity for child language development.

In addition to maternal sensitivity, we aimed to investigate maternal speech components that are considered part of sensitive synchronous communication and were previously shown to be associated with language development. Maternal sensitivity is theorized as containing two parameters—the positive elements in different modalities (gaze, affect, vocal, and tactile) and the real-time coordination of these elements with the infant’s signals (Feldman et al., 2009). Infant directed speech (IDS) which is part of a multimodal, synchronous communication style used with infants to sustain interactions and highlight messages, can be thought of as the positive element in the speech modality (Saint-Georges at al., 2013). However, using IDS without adjustments to infants’ reactivity and preferences is not sufficient for synchronous sensitive interactions. The other component is the real-time coordination and adjustment of caregivers’ speech to the infants’ cues, which supports conversation-like turn-taking exchanges (Saint-Georges et al., 2013). In the current study, these components of early parental speech input, IDS and CTs, and their relation to children’s later language development were directly examined.

Infant-directed-speech is a speech style that exhibits universal prosodic features (e.g., Grieser & Kuhl, 1988; Kuhl et al., 1997). It is characterized by a higher pitch, slower tempo, and exaggerated intonation contours when compared to standard adult-directed speech (Fernald & Simon, 1984). From birth, infants prefer listening to the IDS speech register (Cooper & Aslin, 1990; Fernald, 1985). Infant-directed speech assists in the acquisition of a phonological system by delivering information in an exaggerated form. The phonetic units are clearer, longer, and more distinct from one another compared to standard speech (Kuhl et al., 1997). Previous studies underscore the importance of IDS for child language development (Golinkoff et al., 2015). For example, it was suggested that IDS prosody facilitates young infants’ word segmentation from fluent speech (Bosseler et al., 2013; Thiessen et al., 2005), speech discrimination (Liu et al., 2003; Trainor & Desjardins, 2002), and separation of stimuli with properties of IDS from background noise (Colombo et al., 1995).

The importance of IDS specifically during the early developmental period is further indicated in a study showing that IDS enabled 21-month-old children to learn new words that they did not learn when presented in adult-directed speech (ADS) (Ma et al., 2011). In contrast, 27-month-old children were able to successfully learn new words presented in ADS. The authors suggest that these results support the assumption that IDS facilitates word mapping at the start of lexical acquisition and that its influence decreases as language development proceeds (Ma et al., 2011). A study with 7- and 8-month-old infants examined long-term memory for words that were spoken in either IDS or ADS. They found that word recognition was successful for words introduced in IDS, but not for those introduced in ADS (Singh et al., 2009).

Several studies have explored natural speech input in the home environment and its relation to concurrent and later language development. It was found that Spanish-learning infants who experienced more IDS measured at 19 months of age had larger expressive vocabularies by 24 months (Weisleder & Fernald, 2013). Similarly, a study with English-learning infants at 11- and 14-months found that the amount of exposure to IDS in a 1:1 social context (speech directed to the infant while they are alone with the speaker), but not IDS in a group social context (one or more adults with the infant) was strongly related to the child’s developing language skills at 24 months (Ramírez-Esparza et al., 2014) and persisted at 33 months of age (Ramírez-Esparza et al., 2017a). The same pattern was replicated in a sample of Spanish–English bilingual families (Ramírez-Esparza et al., 2017b). The results showed that the amount of exposure to IDS speech in a 1:1 context (regardless of the language spoken) was related to concurrent vocalizations and overall productive vocabulary (the total number of words produced in both Spanish and English) at 24 months of age (Ramírez-Esparza et al., 2017b).

Another important type of speech element that has been shown to be related to language development is parent–child conversational turns. CTs refer to the back-and-forth vocal exchanges between the child and an adult, which have a key role in children’s language learning (Donnelly & Kidd, 2021; Ferjan Ramírez et al., 2020; Gilkerson et al., 2018; Levinson, 2016; Zimmerman et al., 2009) and neural language processing (Huber, Ferjan Ramírez, et al., 2023; Romeo et al., 2018). For example, a study that collected audio data in the home environment using digital first-person perspective recordings found that the number of adult–child CTs are positively associated with language development measurements at the age of 5 years (Zimmerman et al., 2009). Similarly, a recent study aimed to test the directionality of the relation between CT and early language development in children acquiring English who were followed between 9 and 24 months of age found that the two variables mutually influence each other across early development, supporting arguments for a social basis to early language development (Donnelly & Kidd, 2021). It was further shown that adult–child CTs at 18 to 24 months of age predict school-age language and cognitive outcomes (Gilkerson et al., 2018). The link between CTs and language development was recently investigated in earlier stages of development. CTs assessed at 4- to 6 months of age during mother-infant face-to-face interaction were found to be positively associated with expressive vocabulary skills at 24 months (Nguyen et al., 2023). The importance of CT is further indicated in a cohort of infants having risk factors associated with poorer language development where fluency and connectedness of mother–child conversations at 12 months predicted later language scores at 24 and 36 months of age (Smith et al., 2018). Moreover, in a study that explored causal effects between parental language input and children’s language outcomes, mothers who were randomly assigned to receive a coaching intervention to enhance parental language input increased their use of IDS and were engaged with their child in more CTs compared to mothers who did not receive coaching (Ferjan Ramírez et al., 2020). Their children, in turn, increased their production of speech-related vocalizations significantly more between 6 and 18 months of age, and produced more words at 18 months of age, when compared with the control group whose parents were randomly assigned to the non-coached control group (Ferjan Ramírez et al., 2020). Moreover, the intervention revealed positive effects on expressive language development, through 30 months of age, a full year after the final intervention session (Huber, Ferjan Ramírez, et al., 2023).

The foregoing studies suggest that various characteristics of mother–infant social interaction, such as parental sensitivity and speech style—particularly IDS and CTs—play important roles in language development. Although mothers use IDS and naturally “synchronize” maternal behaviors with their infant’s cues and engage in conversation-like exchanges with their infant by the third month of life (Gratier et al., 2015; Hilbrink et al., 2015; Stern et al., 1975), there are little data on the potential link between speech input prior to 6 months of age and later language development. We hypothesize that early social interactions that exhibit maternal sensitivity, IDS and CTs, enhance infants’ interest and attention to speech and support language acquisition when period of phonetic and word learning starts. The goal of the present study is to empirically examine the relation between mother–infant 1:1 social interaction measures—including speech input (IDS and CTs) and maternal sensitivity at 3 months of age—and subsequent language growth, in a longitudinal study. More specifically, we explored measures of mother–infant interactions in a 1:1 context, because previous studies demonstrated that engaging in 1:1 interactions (vs. group social interactions) in infancy has important implications for language development. It was suggested that 1:1 context allows social contingencies between the adult and child (Ramírez-Esparza et al., 2014, 2017a, 2017b). To assess the effect of both *context* and *speech style*, we also measured maternal IDS in a group context (as opposed to 1:1) as well as maternal standard speech (as opposed to IDS) in 1:1 context. We hypothesized that similar to previous studies (Ramírez-Esparza et al., 2014), these measures would *not* be associated with later productive language outcomes during this early developmental window. In addition, we measured standard speech within a group context, hypothesizing this measure would be negatively correlated with language development scores.

We sought to explore how speech-input scores obtained from the LENA home recordings at 3 months of age are linked to productive language development from 18 to 30 months of age. We note that because infants only begin to engage in conversation-like exchanges from the third month of life, we anticipated a relatively low CT score range at that age; however, we explored whether mother–infant CTs at this early age is related to later language development.

To further explore maternal behavior during mother–infant 1:1 interaction at 3 months of age and its relation to language development, we recorded mother–infant face-to-face interactions in the lab for later offline coding using the well-validated Coding Interactive Behavior (CIB) coding system (Feldman, 1998) and examined whether maternal sensitivity during face-to-face interaction at 3 months of age is related to infants’ prospective language development.

In sum, two specific hypotheses were formulated. First, we expected that both IDS and CTs during mother–infant 1:1 interaction in this early developmental window (3 months of age) would be positively associated with later productive language outcomes. Second, we hypothesized that overall maternal sensitivity would be positively related to language development scores. Finally, in a further, more exploratory data analyses, we aimed to uncover the specific components of the maternal sensitivity construct that are related to later language development.

## 2. Methods

### 2.1. Participants

40 mother–infant dyads from monolingual English-speaking households were recruited to participate in the longitudinal study (infants’ mean age at the first meeting = 76.1 ± 6.7 days; 23 females; mothers’ mean age at the first session = 33.0 ± 3.1 years). Mothers and infants lived in families that varied in socioeconomic status (SES), Hollingshead, 1975; *M* = 54.6 ± 9.0, range = 19 to 66). The inclusion criteria included the following: (a) full term and born within 14 days of the due date, (b) no known health problems and no more than 3 ear infections, (c) birth weight ranging from 6 to 10 lb, (d) no significant foreign language exposure (i.e., parents and regular caregivers speak only English to the infant) and (e) all of the mothers in the study were first time parents. All experimental procedures were approved by the University of Washington Institutional Review Board and all participating families gave informed consent and were compensated monetarily for their time and effort.

### 2.2. Design and procedure

The experimental design included three parts employing three widely used and validated tools, as summarized in Table 1. First, to directly assess behavioral measures of mother–infant 1:1 interaction, the mothers and the infants were invited to the lab and participated in a free play interaction session in the lab. The mothers were instructed to interact naturally with their infants for at least 3 min in a 9 ft x 10 ft room. The room had three cameras and a microphone to capture the mother’s and infant’s face and body. The mothers were asked to begin the interaction seated on the floor facing one of the cameras with their infant lying down on their back on a blanket in front of them. The mothers were instructed to stay on the floor, but that they could move their baby once the recording begins. A limited number of toys with a variety of functions (e.g., a ball, a rattle, a pinwheel, a baby doll, stacking cups) were available in the room for the mothers to use, but they were not instructed to do so. The infant and the mother were left alone in the experiment room during the interaction. The interactions were videotaped for later offline coding using the CIB manual (Feldman, 1998).

Second, within one week following the in-lab interaction session, the families completed LENA recordings in their home environment for assessment of language input (infants’ mean age = 82.2 ± 8.9 days). Third, language development was assessed when the infant participants reached the age of 18 months and continued 30 months of age using the MacArthur-Bates Communicative Development Inventory (CDI) (Fenson et al., 1994), collected in 3-month intervals (18, 21, 24, 27, and 30 months of age).

#### 2.2.1. Coding interactive behavior manual (CIB)

To assess behavioral measures of the mother–infant free-play 1:1 interaction recorded in the lab, we used the CIB manual (Feldman, 1998). The CIB is a global rating system of social interaction and is comprised of multiple scales scored from 1 (low) to 5 (high) that combine into theoretically-derived constructs. The CIB has been validated in a large number of studies spanning over 25 countries in healthy and high-risk populations. The system has shown construct validity, test-retest reliability, and prediction to multiple social, cognitive, biological, and neural outcomes (Feldman, 2012, 2021). The CIB utilizes scales for parent, for infant, and for the dyadic atmosphere.

The most widely-reported maternal behavioral constellation is “maternal sensitivity.” The sensitivity construct of the CIB has been used in a large number of studies in infancy and showed individual stability from infancy to adolescence (Feldman, 2010), and prediction to positive and negative social–emotional outcomes, including mental health, stress, affiliation hormones (Feldman & Eidelman, 2009; Pratt et al., 2019; Ulmer-Yaniv et al., 2018; Yirmiya et al., 2018, 2020), and brain activation patterns (Zeev-Wolf et al., 2019, 2022). Maternal sensitivity included the following codes: mother acknowledgment, maternal vocalization, positive affect, maternal gaze and mother affectionate touch. A coder, trained to 85% reliability and blind to all other information, coded the interactions.

#### 2.2.2. Language environment analysis system, LENA

Similar to previous studies (Ferjan Ramírez et al., 2020; Ramírez-Esparza et al., 2014, 2017a) investigating the relation of IDS input to language development, we used the LENA system (Language Environment Analysis Foundation, Boulder, Colorado) to assess everyday social interactions between adults and infants in natural settings over two consecutive days.

LENA devices are designed to capture the naturalistic language environment of children. The LENA recorder can store up to 16 h of digitally recorded sound and can be snapped into a pocket on the front of a child’s vest. This allows the recorder to unobtrusively capture all language in the child’s environment. The recordings can be subsequently downloaded and analyzed by LENA software. The LENA software provides several automated measures of the language content, but for the purposes of the present study, all language measures were coded manually. The LENA audio files were processed using the LENA Advanced Data Extractor Tool (ADEX) to automatically identify segments for further manual analyses. Each participant’s two daily recordings were segmented into 30-seconds intervals. To identify intervals with language activity for each of the 2 recording days, 50 intervals with the highest number of adult words heard by the child (AWC) that were at least 3 min apart were automatically selected, yielding a total of 100 30-seconds coding intervals per participant. Coders listened to each 30 s interval and entered a “Yes” or a “No” for each of the following coding categories: (a) Mother infant-directed speech (IDS)– 1:1, (b) Mother infant-directed speech (IDS)-group, (c) Mother Standard Speech-1:1, (d) Standard Speech-group and (e) Mother–Infant CTs-1:1. Definitions used for manual coding are provided in Table 1. The resulting matrix of Yes and No responses for each 30 s interval indicated that a specific category occurred or did not occur in that interval. For clarification, for Mother IDS-1:1, any segment that contains Mother IDS in 1:1 context is counted as “Yes,” even if it’s followed by standard adult-directed speech. This means that segments counted as containing Mother IDS-1:1 may also contain other types of speech inputs from the mother. Then the percentage of intervals coded for each category were calculated: the total percentage of intervals with mother IDS-1:1 (%Mother IDS-1:1), mother IDS in a group (%Mother IDS-group), mother standard speech-1:1 (%Mother Standard Speech-1:1), standard speech-group (%Standard Speech-group), and mother–infant CTs-1:1 (%Mother-Infant CTs-1:1).

#### 2.2.3. MacArthur-bates communicative development inventory (CDI)

Productive vocabulary was assessed when participants reached the age of 18 months and continued at 3-month intervals to 30 months of age using the CDI. In the current study, we used CDI percentile scores. Percentile scores provide individual’s relative position in comparison to a large norming sample for boys and girls. Two specific measures were assessed:

##### (a) Vocabulary percentile scores:

Vocabulary is measured by parents’ checking all the words the child can produce on a word list on the survey, with a maximum number of 680 words.

##### (b) Irregular words percentile score:

The irregular word section contains a checklist of 25 irregular nouns and verbs (e.g., teeth, got). Children’s early usage of correct irregular word forms is one of the first signs that they are learning the morphology of their language. This section of the inventory asks parents to specify whether the child has begun to use each of five common irregular plural nouns and 20 common irregular past-tense verbs. Although some children produce irregular forms in their second year, it is in the third year that acquisition of irregular morphology accelerates (Fenson et al., 1994).

### 2.3. Exclusion data and power analysis

One dyad was not included in the analysis since the parents failed to complete the CDI forms. Four additional participants were excluded because their vocabulary output scores at 30 months were lower than 2.5 standard deviations from the group mean (less than 318 words, group mean following exclusion = 570.5 ± 100.7 words). One additional participant was excluded from further analysis because their %Mother IDS-1:1 exceeded more than 2.5 standard deviations from the group mean (more than 50%, group mean following exclusion = 24.2 ± 10.5%). All further analysis with LENA and CDI measurements involved 34 mother–infant dyads. In addition, one dyad was excluded from the CIB analysis since the infant showed extreme withdrawal behavior.

To estimate needed sample size, we conducted power analysis using SPSS software based on a previous study (Ramírez-Esparza et al., 2017b) that used similar methods (LENA and CDI) and variables (speech input and language development) and reported a significant positive correlation (*r* = .49) between IDS speech-1:1 and later productive vocabulary. Using an alpha of 0.05 and a power of 0.80, the required sample size was determined to be at least 26 participants.

## 3. Results

### 3.1. Language input and maternal sensitivity at 3 months and relations to productive language development from ages 18 to 30 months

We sought to explore how speech input scores obtained from the LENA home recordings at 3 months of age were linked to CDI productive language development at 18, 21, 24, 27 and 30 months of age. The LENA measures used in the analysis including: (a) *% Mother IDS-1:1* (Percentage of intervals, mean = 24.2, *SD* = 10.6, median = 24.0, range = 4 – 49), (b) *%Mother IDS-group* (Percentage of intervals, mean = 18.7, *SD* = 7.4, median = 18.5, range = 4 – 34), (c) *%Mother Standard Speech-1:1* (Percentage of intervals, mean = 9.5, *SD* = 6.7, median = 7.5, range = 0 – 22), (d) *%Standard Speech-group* (Percentage of intervals, mean = 23.5, *SD* = 10.0, median = 22.0, range = 4 – 47) and (e) *%Mother*–*Infant CTs-1:1* (Percentage of intervals, mean = 2.2, *SD* = 2.7, median = 2.0, range = 0 – 11). To further explore maternal behavior during mother–infant 1:1 interaction at 3 months of age and its relation to language development, we used the CIB scores obtained from the mother–infant free play face-to-face interactions to examine whether *Maternal sensitivity* (mean = 3.8, *SD* = 0.5, median = 4.0, range = 2.6 – 4.6), is also related to infants’ language development. The speech input measurements as well as sensitivity histograms can be found in Supplementary Fig. S1.

**Fig. 1.**
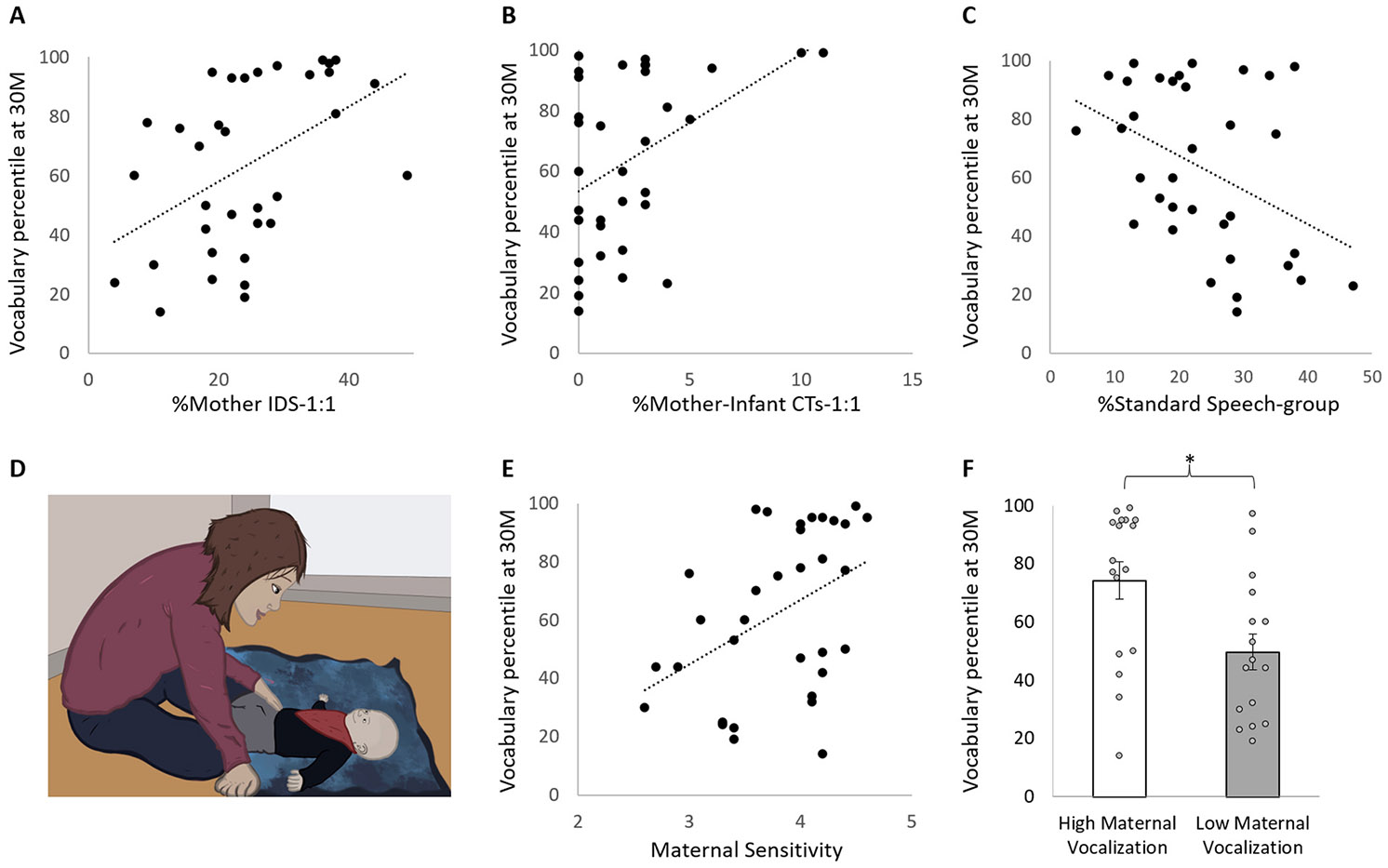
Speech input at 3 months of age and language outcomes at 30 months of age. Scatter plots showing the relationships between (A) %Mother IDS-1:1 at 3 months of age and vocabulary percentile scores at 30 months of age, (B) %Mother–Infant CTs-1:1 at 3 months of age and vocabulary percentile scores at 30 months, (C) %Standard Speech (adult directed speech) within a group at 3 months of age and vocabulary percentile scores at 30 months (D) illustration of mother–infant face-to-face interaction at the lab. (E) Maternal sensitivity during face-to-face interaction at 3 months of age and vocabulary percentile scores at 30 months of age (F) Mean ± 1 SEM vocabulary percentile scores at 30 months of age in two subgroups: high maternal vocalization (*n* = 17) and low maternal vocalization (*n* = 16). Non-parametric Mann Whitney test revealed significant differences in vocabulary percentile scores (*p* = 0.01), with higher vocabulary percentile scores in the “high maternal vocalization” subgroup (mean vocabulary percentile scores = 74.2 ± 26.4%) compared with “low maternal vocalization” subgroup (mean vocabulary percentile scores = 49.7 ± 24.5%). *p < 0.05.

Initially, intercorrelations among study variables were examined using Spearman correlations (Table 2). The analysis revealed that %Mother IDS-1:1 at 3 months of age is significantly and positively correlated with vocabulary percentile scores at 24, 27, and 30 months of age (*n* = 34, all *r*_s_ > 0.45, all *p*s < 0.01, see Fig. 1A for %Mother IDS-1:1 correlation with vocabulary percentile scores at the oldest age measured, 30 months of age). The strongest correlation occurred at 27 months of age (*r*_s_ = 0.51, *p* = 0.002). No significant correlations were found between %Mother IDS-group and vocabulary percentile scores at any time point (all ∣*r*_s_∣ < 0.2, all *p*s > 0.25), or between %Mother Standard Speech-1:1 and vocabulary percentile scores at any time point (all ∣*r*_s_∣ < 0.2, all *p*_s_ > 0.3). However, % Standard Speech-group was *negatively* associated with vocabulary percentile scores at 21, 24, 27, and 30 months of age (all *r*_s_ < −0.34, all *p*s < 0.05, see Fig. 1C. for % Standard Speech-group correlation with vocabulary percentile scores at the oldest age measured, 30 months of age) (Table 2).

At 3 months of age, %Mother–Infant CTs-1:1 was significantly and positively associated with infants’ vocabulary percentile scores at 27 and 30 months of age (*n*= 34, both *r*_s_ > 0.37, both *p*s < 0.05, see Fig. 1B for %Mother-Infant CTs-1:1 correlation with vocabulary percentile scores at the oldest age measured, 30 months of age). Notably, the amount of CTs at 3 months of age is relatively small, with no recorded Mother-Infant CTs-1:1 for 12 dyads.

Maternal sensitivity at 3 months of age during mother–infant face-to-face interaction recorded in the lab (see Fig. 1D. for illustrated interaction) was associated with infants’ vocabulary percentile scores at 18, 21, 24, 27 and 30 months of age (N = 33, all *r*_s_ > 0.35, all *p*s < 0.05, see Fig. 1E. for the correlation between maternal sensitivity at 3 months of age and vocabulary percentile scores at the oldest age measured, 30 months of age), with the strongest correlation occurring at 27 months of age (*r*_s_ = 0.49, *p* = 0.004).

To check the obtained results, we repeated the same analysis exploring the relation between speech input variables and maternal sensitivity to CDI productive language development using Vocabulary Raw scores, and obtained the same pattern of results (see Supplementary Table S1).

Previous investigations have reported links between socioeconomic status (SES) and language development (e.g., Fernald et al., 2013; Hart & Risley, 1995) and between SES and IDS speech in the context of 1:1 interaction (Ramírez-Esparza et al., 2014). However, in the current study we found no significant correlation between SES and any of the variables (all ∣*r*_s_∣ < 0.3, all *p*s > 0.1), thus we did not control for SES in the main text, but to further check the main results, we repeated the same analysis controlling for SES (see Supplementary Table S2) and we obtained substantially the same pattern of results.

Because the two speech input variables, %Mother IDS-1:1 and %Mother-Infant CTs-1:1, are significantly correlated (*r*_s_ = 0.45, *p* = 0.007), as well as Maternal sensitivity and %Mother-Infant CTs-1:1 (*r*_s_ = 0.43, *p* = 0.013), we further explored the obtained correlations accounting for the effect of the other variable using partial Spearman correlations. The analysis revealed no significant contribution for %Mother-Infant CTs-1:1 beyond %Mother IDS-1:1 or Maternal sensitivity in the association with vocabulary percentile scores for any of the measured time points (%Mother-Infant CTs-1:1 conditioned on %Mother IDS-1:1, all *r*_s_ < 0.25, all *p*s > 0.17, %Mother-Infant CTs-1:1 conditioned on Maternal sensitivity, all *r*_s_ < 0.21, all *p*s > 0.25). However, a partial Spearman correlation revealed a significant contribution for %Mother IDS-1:1 in the association with vocabulary percentile scores at 24, 27 and 30 months of age (%Mother IDS-1:1 conditioned on %Mother-Infant CTs-1:1, all three time points *r*_s_ > 0.37, all *p*s < 0.035). Similarly, the analysis revealed a significant contribution for Maternal sensitivity in the association with vocabulary percentile scores at 18, 24 and 27 months of age (Maternal sensitivity conditioned on %Mother-Infant CTs-1:1, all three time points *r*_s_ > 0.36, all *p*s < 0.045) and the two other time points had trends toward positive correlations (both at 21 and 30 months of age, *r*_s_ > 0.33, *p*s < 0.063).

In addition to vocabulary percentile scores, we also used another CDI variable, the irregular words percentile score. Children’s early usage of correct irregular word forms is one of the first signs that they are learning the morphology of their language. We repeated the same analysis with infants’ irregular words percentile scores and obtained similar results (Supplementary, Table S3).

### 3.2. Infant-directed speech and maternal sensitivity: contributions to language development

Finally, a regression analysis was used to assess the relative contribution of early speech input, including %Standard Speech-group and %Mother IDS-1:1, and Maternal sensitivity at 3 months of age on vocabulary percentile scores at 30 months of age. Since %Mother IDS-1:1 and %Mother-Infant CTs-1:1, as well as Maternal sensitivity and %Mother-Infant CTs-1:1, are intercorrelated, and partial correlations controlling for %Mother-Infant CTs-1:1 revealed a significant contribution for %Mother IDS-1:1 and Maternal sensitivity in the association with language development scores, we did not include %Mother-Infant CTs-1:1 in the model.

The overall model predicted approximately 34.9% of the variance in vocabulary percentile scores at 30-months of age (*R*^*2*^ = 0.41, *F* (3,29) = 6.7, *p* = 0.001). The results of the regression indicated that %Mother IDS-1:1 significantly predicted vocabulary percentile scores at 30 months of age (*t* = 2.3, *p* = 0.032), as did Maternal sensitivity during mother–infant 1:1 interaction (*t* = 2.2, *p* = 0.034), while %Standard Speech-group showed a nonsignificant *negative* association with vocabulary percentile scores (*t* = −1.9, *p* = 0.062) (see Table 3).

Similarly, a regression analysis was used to assess the relative contribution of early speech input and Maternal sensitivity at 3 months of age on irregular words percentile scores at 30 months. The overall model predicted approximately 42.1% of the variance in irregular word percentile scores at 30 months of age (*R*^*2*^ = 0.48, *F*(3,29) = 8.8, *p* < 0.001). The results of the regression indicated that %Mother IDS-1:1 significantly predicted irregular words percentile scores at 30 months of age (*t* = 3.3, *p* = 0.002), as did Maternal sensitivity during mother–infant 1:1 interaction (*t* = 3.0, *p* = 0.006), while %Standard Speech-group did not (*t* = −0.5, *p* = 0.60) (Table 3).

### 3.3. Decomposition of maternal sensitivity variable to its components and exploring their relation to later language development

To further explore the detected association between Maternal sensitivity and language development scores, we tested each of the Maternal sensitivity components separately and looked for associations between these measurements and vocabulary percentile scores. Maternal sensitivity is composed of maternal vocalization, mother acknowledgment, positive affect, mother gaze and mother affectionate touch.

While affectionate touch was not intercorrelated with any of the other maternal sensitivity components (all ∣*r*_s_∣ < 0.12, all *p*s > 0.5), all the other components were significantly intercorrelated with each other (all *r*_s_ > 0.49, all *p*s < 0.005). Affectionate touch was significantly associated with vocabulary percentile scores at 21, 24, and 27 months of age (all 3 time points *r*_s_ > 0.35, all 3 time points *p*s < 0.05).

Partial Spearman correlation revealed no significant contribution for maternal gaze, acknowledgment or positive affect beyond the other variables in the association with vocabulary percentile scores for all measured time points (conditioned on other three intercorrelated components, all *r*_s_ < 0.32, all *p*s > 0.09). However, a partial Spearman correlation revealed a significant contribution for maternal vocalization in the association with vocabulary percentile scores at 21, 24, 27 and 30 months of age (maternal vocalization conditioned on positive affect, gaze and acknowledgment, all 4-time points *r*_s_ > 048, all *p*s < 0.007).

To further explore the role of maternal vocalization during the 3-minutes of face-to-face free play interaction, we split the dyads into two subgroups according to the maternal vocalization median score (median = 5) resulting with a group of 17 dyads with a score of 5 and 16 dyads that scored below 5 (mean = 3.6, *SD* = 0.43, median = 3.75, range = 3 - 4). We then compared the vocabulary percentile scores between the two subgroups using the non-parametric Mann Whitney test. The analysis revealed significant differences in vocabulary percentile scores between the “low vocalization” and the “high vocalization” subgroups in all time points (all *p*s < 0.05), with higher vocabulary percentile scores in the “high vocalization” group (Fig. 1F for vocabulary percentile scores at 30-months).

## 4. Discussion

The goal of the current study was to examine the associations between one-on-one (1:1) interaction at a very early window in development, 3 months of age, and measures of productive language development to the age of 30 months of age. In addition, we also examined language input at home using LENA recordings and related these measures to language outcomes to the age of 30 months. Several important and novel findings are shown by the data.

### 4.1. Early Maternal sensitivity and prospective language development

We found that maternal sensitivity measured in the laboratory during mother–infant interaction at 3 months of age is significantly associated with infants’ productive language development at multiple time points, from ages 18 to 30 months, with both vocabulary and irregular words percentile scores. Maternal sensitivity is the ability to respond appropriately to the child’s social cues, that is, the coordination of maternal behavior with infant signals. Parental sensitivity has long been shown to have a role in cognitive and social development (e.g., Madigan et al., 2019; Belsky & Pasco Fearon, 2002; Feldman et al., 2004), including language development. Our findings fit together well with previous studies in infants older than 5 months of age showing the importance of sensitive and responsive caregiving for infants’ communicative development, including both real-time vocalization modifications and long-term effects on language acquisition (Baumwell et al., 1997; Bornstein et al., 2020; Brookman et al., 2023; Madigan et al., 2019; Nicely et al., 1999; Tamis-LeMonda et al., 2001; Warren et al., 2010). The results reported here suggest that individual differences in maternal sensitivity at a very early window in development, 3 months of age, may play a significant role in language development. An interesting finding in the current study is that Maternal sensitivity is associated with %Mother–Infant CTs-1:1 but not with %Mother IDS-1:1. This makes sense because in contrast to maternal sensitivity, an intrusive style can often be expressed in excessive parental behavior that disregards the child’s signals (Kaitz & Maytal, 2005). For example, it was previously reported that anxious mothers maintained high-pitched, sing-song vocalizations for much of the mother–infant interaction, regardless of whether the infant was socially responsive, gaze averting, or showing signs of fatigue (Feldman et al., 2007a, 2009).

Our finding that maternal sensitivity was associated with CTs, but not with IDS highlights the importance of real-time coordination as a crucial component in sensitive behavior, and while use of positive vocalization is ordinarily a component of sensitive caregiving, it is not sufficient if the stimuli are not adjusted to infants’ signals, which enables the infants to respond and communicate as well. We speculate that as infants continue to develop and become more actively engaged in communication, the correlation between sensitivity and the level of CTs may even strengthen. Exploring the contribution of each of the components of maternal sensitivity to the obtained correlations revealed that the most significant component is maternal vocalization. According to the CIB coding system, high maternal vocalization scores represent warm, clear, IDS vocalizations that are adapted to the infant’s cues.

Maternal affectionate touch was also significantly correlated with language outcomes. In humans, 65% of face-to-face interactions between mothers and infants involve touch communication (Stack & Muir, 1990). Affectionate touch plays a unique and vital role in infant development. Previous studies have established that maternal affectionate touch during the postpartum period is associated with wide-ranging benefits for infant neurobehavioral, cognitive and social-emotional development (Cascio et al., 2019; Carozza & Leong, 2021; Feldman & Eidelman, 2003; Feldman et al., 2010), and also that young infants show brain responses exquisitely timed to being touched (Meltzoff et al., 2018, 2019). The contribution of touch for language acquisition is also indicated in a study showing that experimenter-provided synchronous tactile cues help 4-month-olds to find words in continuous speech. In the study, caregiver touch paired with concurrent speech was shown to play an important role in infants’ ability to detect word boundaries. The authors suggest that contingent touch may be a powerful mechanism that enhances attention and can provide information for word-learning in children (Seidl et al., 2015).

### 4.2. Early speech input and future language development

We found that IDS during mother–infant 1:1 interaction in the home environment at 3 months of age is significantly correlated with language development scores (both vocabulary and irregular words percentile scores) at 24, 27, and 30 months of age. The importance of IDS during infancy for concurrent and future language development has been previously highlighted (Golinkoff et al., 2015; Ma et al., 2011; Ramírez-Esparza et al., 2014, 2017a; Singh et al., 2009; Thiessen et al., 2005; Trainor & Desjardins, 2002). However, to our knowledge this is the first study demonstrating that the quality of speech input during a very early window in development, 3 months of age, is significantly related to later language development.

To assess the potential contribution of the context of the interaction, we further explored maternal IDS in a group context (in contrast to IDS in a 1:1 context). In line with previous studies (Ramírez-Esparza et al., 2014, 2017a, 2017b), we found no correlation between IDS in a group context and prospective language development scores. This finding emphasizes the special quality of caregiver–infant 1:1 interactions and their role in supporting language development. It was previously suggested that the pattern that caregivers establish in 1:1 interaction with their children during infancy is maintained in childhood, and although caregivers change speech style across time, the social context is maintained and highlights the importance of contingent bi-directional interactions on language development (Ramírez-Esparza et al., 2017a). We speculate that during parent-infant 1:1 interaction, as opposed to group settings, infants experience more responsive social feedback. In addition, we measured %Mother Standard Speech-1:1, and as expected, we did not find a significant correlation between this speech variable and language development scores. In contrast to IDS in 1:1 context, standard speech within a group is negatively correlated with infants’ vocabulary percentile scores from ages 21 to 30 months, but not with irregular words scores. These findings suggest that both the speech style and the context of the interaction are important factors in the observed correlation between speech input and language development.

It should be noted that significant correlations between %Mother IDS-1:1 and language development scores were found at 24, 27, and 30 months but not for younger ages of 18 and 21 months. Between 18 and 24 months old, children typically undergo significant language development. The rate of early vocabulary development has been described as slow in the first few months after the emergence of first words followed by a period of accelerated growth, sometimes termed the “vocabulary spurt” or “naming explosion” (Fenson et al., 1994; Goldfield & Reznick, 1990). Although language development scores show strong intercorrelation across the five measured time points (all *p*s < 0.001), it is possible that individual differences in vocabulary scores before 24 months of age may not be fully captured by the measures used in the current study. Additionally, the absence of significant correlations may be attributed to a comparatively low variability of vocabulary percentile scores during the early age. Interestingly, although correlations with %Mother IDS-1:1 were significant for language development starting at 24 months of age, maternal sensitivity showed a significant correlation with language development scores across all measured time points, from 18 to 30 months. That being said, similar to %Mother IDS-1:1, the strongest correlation with vocabulary scores was observed at 27 months of age. The maternal sensitivity construct includes a diverse range of parental behaviors across various modalities, coordinated with infants’ signals, and is not specific to speech. We speculate that this multimodal combination is important for development and thus may be involved in the differences in vocabulary scores even before 24 months as indicated by correlations with vocabulary percentile scores across all measured time points.

Similar to %Mother IDS-1:1, we found that %Mother–Infant CTs-1:1 at 3 months of age are significantly associated with infants’ productive language development scores at 27 and 30 months of age. At this early age conversational turns were only initiated by the caregiver. Although the effect was significant, the correlation between CTs and productive vocabulary was influenced by a relatively small number of dyads and therefore should be interpretated with caution. Nonetheless, this link between CT and language development is supported by previous research with older infants, which indicates a key role for CTs in children’s language learning (Donnelly & Kidd, 2021; Ferjan Ramírez et al., 2020; Gilkerson et al., 2018; Levinson, 2016; Zimmerman et al., 2009).

We further found a significant contribution for %Mother IDS-1:1 that went beyond %Mother–Infant CTs-1:1 with both vocabulary and irregular words percentile scores. However, when we controlled for %Mother IDS-1:1, the effect was no longer significant for % Mother–Infant CTs-1:1. These data suggest that IDS plays a significant role in language development, regardless of whether the caregiver engages in conversational turns. Support for this can be found in a study comparing infants of insensitive mothers with intrusive styles (overstimulating behavior) and withdrawn styles (understimulating behavior). Intrusive behavior can be characterized by loud, non-contingent speech and exaggerated, “fake” facial expressions, while withdrawal can be characterized by rare touching, flat affect, and minimal speech throughout the interaction. Infants of mothers with withdrawn style compared to those of intrusive style showed less optimal interactive behavior, including less orientation toward the mother, more gaze aversion and less physical activity (Jones et al., 1997). It was further suggested that less stimulation may contribute to slower language development (Jones et al., 1997). We speculate that infants’ initial preference for IDS enhances their attention to social interaction. We propose that at this early developmental stage IDS provides the foundation for infants’ future active engagement in social interaction and communication and supports language development. This result further underscores the caregiver’s pivotal role in engaging the infant in social interaction during this early developmental window, preceding the 6- to 12-month-old window of phonetic and word learning (e.g., Kuhl et al., 2006, 2010; White et al., 2013).

### 4.3. Summary and future directions

Our current results revealed that early individual differences in social verbal interactions and in maternal sensitivity at 3 months of age, prior to the well-documented periods for phonetic and word learning, are significantly associated with subsequent language growth. Recent studies have explored the neural mechanism of mother–infant face-to-face interaction. These studies show that maternal sensitivity during mother–infant face-to-face interaction is associated with higher mother–infant neural synchrony specifically over infants’ temporal regions, while intrusiveness is associated with diminished interbrain synchrony (Endevelt-Shapira & Feldman, 2023). In contrast, intrusive parenting describes a pattern of care that correlates with maternal anxiety and is linked with negative child outcomes, both in the socio-emotional domain and in terms of more general cognitive development, including language development (Conway et al., 2018; Keown et al., 2001; Nelson, 1973). Parental intrusiveness is often expressed in excessive parental behavior that disregards the child’s communications and provides stimulation when the infant signals a need for rest (Kaitz & Maytal, 2005). Intrusiveness is mostly observed during parent–infant play and refers to the parent’s tendency to control the situation and to interfere with or override the infant’s activities rather than follow the infant’s “lead” in a socially contingent, real-time adjusted fashion (Egeland et al., 1993; Huffmeijer et al., 2020) and that such behavior reduces children’s verbal response opportunities, potentially disrupting language learning. It was suggested that a possible neural basis for such disrupted language development is the reduced left-frontal to right-temporal interbrain synchrony, which taps regions involved in language production and perception (Endevelt-Shapira & Feldman, 2023). In contrast to intrusiveness, sensitive interactions are characterized by reciprocal responsiveness and conversational turns. Along these same lines, an fNIRS hyperscanning study found that more frequent turn-taking during mother–infant face-to-face interaction was related to interpersonal neural synchrony (Nguyen et al., 2023). Such findings also accord with two other studies employing dual-fNIRS and dual-MEG hyperscanning that investigated mother–child verbal turn taking later in development (Lin et al., 2023; Nguyen et al., 2021).

It should also be pointed out that coordinated sensitive interactions may play a role in human social brain development (Endevelt-Shapira et al., 2021; Feldman, 2007a). It has been further suggested that face-focused interbrain synchrony is a human-specific mechanism that uses humans’ inborn face preference to fine-tune the developing social brain (Endevelt-Shapira et al., 2021). Indeed, a recent functional imaging study found that CTs at 6 months of age are predictive of language related white matter pathway maturation at 2 years of age (Huber, Corrigan, et al., 2023). Taken together, we speculate that one pathway by which sensitive mother–infant interactions exert their long-term effect on language development may relate to increased brain-to-brain synchrony which in turn supports social brain maturation, specifically within pathways related to expressive language development. Future research should investigate how maternal sensitivity and speech input variables such as CT and IDS during mother–infant interaction, influence ongoing brain-to-brain synchrony and infant neural responses as early as 3 months of age. It will be especially informative for both cultural and neurobiological theories in developmental science to further examine the mechanisms by which neural synchrony during early sensitive interactions, as well as IDS and CT at 3 months of age, is associated with later language development.

Several study limitations should be acknowledged. First, previous studies suggested that caregivers engage in 1:1 interaction and use IDS with infants who babble more, and are less likely to engage in these behaviors with infants who babble less (Locke, 2006; Ramírez-Esparza et al., 2014). Thus, the obtained relation between speech input and language outcome could reflect, in part, the amount that infants babble at 3 months of age. Future studies should directly measure infants’ vocalizations at 3 months of age and its interaction with maternal speech input and language development.

Second, maternal sensitivity is a caregiving profile which is stable over time. Therefore, maternal sensitivity observed at 3 months of age could possibly reflect maternal sensitivity across infancy. Thus, based on the current study, we cannot conclude that maternal sensitivity at this specific early stage of development, 3 months of age, directly influences later language development. Another possibility is that it is the cumulative effect of experiencing sensitive mothering, beginning as early as 3 months of age, that supports language learning. Future studies should examine maternal sensitivity during mother–infant interactions at more frequent time points throughout development and test their relation to language development, as well as conduct targeted intervention studies more equipped to get at causal mechanisms.

Third, it is important to note that in the current study four subjects were excluded due to low vocabulary scores. In addition, the current study was restricted to first-born infants. Therefore, our findings may not be fully generalizable to the entire population. Future research should follow a larger sample size, including more diversity in the family environments (i.e. number of siblings, strength of support systems, cultural differences in the perceived value of one-on-one interaction, etc.), which would be expected to influence the opportunities for one-on-one interaction.

Finally, we acknowledge that the current study was conducted in a US metropolitan area with families with the financial means and time to visit the laboratory for our assessments. The fact that we uncovered relations between early language input measures and later language performance may not generalize to other cultures. Measures of maternal sensitivity may also vary across cultures, reducing generalizations of our findings. Future studies could adapt the multi-measure longitudinal approach we used here and more frequently assess maternal sensitivity across development and cultures. Such work will allow us to better understand how parent-infant social interactions during the early months of life, far before infants’ first spoken words, potentially influence and support later language development.

## 5. Conclusions

Our results converge to suggest connections between early social interactions and language development. We found that maternal sensitivity during face-to-face interaction at 3 months of age was significantly associated with infants’ productive language development from ages 18 to 30 months. Further, we found that Mother IDS and CTs during mother–infant 1:1 interaction in the home environment at 3 months of age is significantly correlated with prospective measures of language development.

Our current results, along with previous studies showing the importance of caregiver responsiveness, IDS, and back-and-forth conversation exchanges for language development fit together with the reported lack of language learning in the absence of a socially responsive person (Kuhl et al., 2003). These results are consistent with the hypothesis that social interactions and the social brain may “gate" early language learning (Kuhl, 2007, 2011; Lytle & Kuhl, 2017). We have suggested several behavioral and neurobiological contributors that may mediate the relation between early mother-infant interactions and subsequent language development, and suggested needed empirical work that may continue to advance our knowledge about the social contributors to language acquisition.

## Supplementary Material

Mother-infant supp

## Figures and Tables

**Table 1:** Tools and variables used in the current study.

Tool	Age assessed	Variables	Variable definition
Coding Interactive Behavior manual (CIB)	3 months	Maternal sensitivity	Maternal sensitivity is composed of the following codes: mother acknowledgment, maternal vocalization, gaze, positive affect, and mother affectionate touch
Language Environment Analysis system (LENA)	3 months	Mother infant-directed speech (IDS)–1:1	Mother spoke directly to the infant, infant-directed speech style (high pitch, slow tempo, and exaggerated contours) was used, and only the mother’s voice was recorded during the interval
		Mother infant-directed speech (IDS)-group	Mother spoke directly to the infant, IDS speech was used, and two or more adult voices were recorded during the interval
		Mother Standard Speech–1:1	Mother spoke directly to the infant, standard (ordinary) speech was used, and only the mother’s voice was recorded during the interval
		Standard Speech–group	Mother, father, or other adult spoke directly to the infant, standard (ordinary) speech was used, and more than one adult voice was recorded during the interval
		Mother–infant conversational turns (CTs)–1:1	Mother’s utterances followed within 5 s by a child utterance, or vice versa, and only the mother’s voice was recorded during the interval
Communicative Development Inventory (CDI)	18, 21, 24, 27, and 30 months	Vocabulary percentile scores	Parents check all words the child can produce on a word list, with a maximum number of 680 words
		Irregular words percentile scores	Parents check all irregular words the child can produce on a word list. The irregular word section contains a checklist of 25 irregular nouns and verbs

**Table 2:** Correlations between social interaction variables (LENA and CIB) assessed at 3 months of age and vocabulary percentile scores.

Variable	Vocabulary 18 M	Vocabulary 21 M	Vocabulary 24 M	Vocabulary 27 M	Vocabulary 30 M
%Mother IDS-1:1	*r* = 0.28,	*r* = 0.28,	*r* = 0.46,	*r* = 0.51,	*r* = 0.50,
	*p* = 0.10	*p* = 0.11	*p* = 0.007 **	*p* = 0.002 **	*p* = 0.003**
%Mother IDS-group	*r* = −0.06	*r* = −0.20	*r* = −0.08	*r* = −0.03	*r* = 0.008
	*p* = 0.72	*p* = 0.26	*p* = 0.67	*p* = 0.9	*p* = 0.96
%Mother Standard Speech-1:1	*r* = 0.06,	*r* = 0.16,	*r* = 0.17,	*r* = 0.16,	*r* = 0.10,
	*p* = 0.74	*p* = 0.37	*p* = 0.35	*p* = 0.38	*p* = 0.58
%Standard Speech-group	*r* = −0.19,	*r* = −0.35,	*r* = −0.35,	*r* = −0.37,	*r* = −0.37,
	*p* = 0.29	*p* = 0.04 *	*p* = 0.04 *	*p* = 0.03 *	*p* = 0.03 *
%Mother-Infant CTs-1:1	*r* = 0.27,	*r* = 0.21,	*r* = 0.31,	*r* = 0.38,	*r* = 0.41,
	*p* = 0.13	*p* = 0.23	*p* = 0.07	*p* = 0.03 *	*p* = 0.016 *
Maternal Sensitivity (CIB)	*r* = 0.43,	*r* = 0.37,	*r* = 0.42,	*r* = 0.49,	*r* = 0.43,
	*p* = 0.012 *	*p* = 0.036 *	*p* = 0.014 *	*p* = 0.004 **	*p* = 0.012 *

Note: * *p* < .05, ** *p* < .01

**Table 3:** Results of linear regression analysis.

Outcome Variable	Predictor Variable	Beta	*SE*	95 CI%		*β*	*p*
				*LL*	*UL*		
Vocabulary percentile scores	%Mother IDS-1:1	0.89	0.39	0.08	1.69	0.33	0.032 *
	%Standard Speech-group	−0.79	0.41	−1.63	0.04	−0.29	0.062
	Maternal sensitivity	16.7	7.5	1.33	32.14	0.32	0.034 *
Irregular percentile scores	%Mother IDS-1:1	1.54	0.46	0.59	2.5	0.46	0.002 **
	%Standard Speech-group	−0.25	0.48	−1.24	0.73	−0.07	0.6
	Maternal sensitivity	26.5	8.9	8.3	44.7	0.41	0.006 **

Note: *p < .05, ** *p* < .01

## Data Availability

Data will be made available on request.

## Appendix A. Supporting information

Supplementary data associated with this article can be found in the online version at doi:10.1016/j.infbeh.2024.101929.
