# Supplementary material for: Mother–infant social and language interactions at 3 months are associated with infants’ productive language development in the third year of life
Source: Infant Behav Dev. Author manuscript; Available in PMC 2026 Apr 13. (PMC13074446; doi:10.1016/j.infbeh.2024.101929)
Supplement: Mother-infant supp [file NIHMS2155550-supplement-Mother-infant_supp.docx]

**Supplementary Material**

**
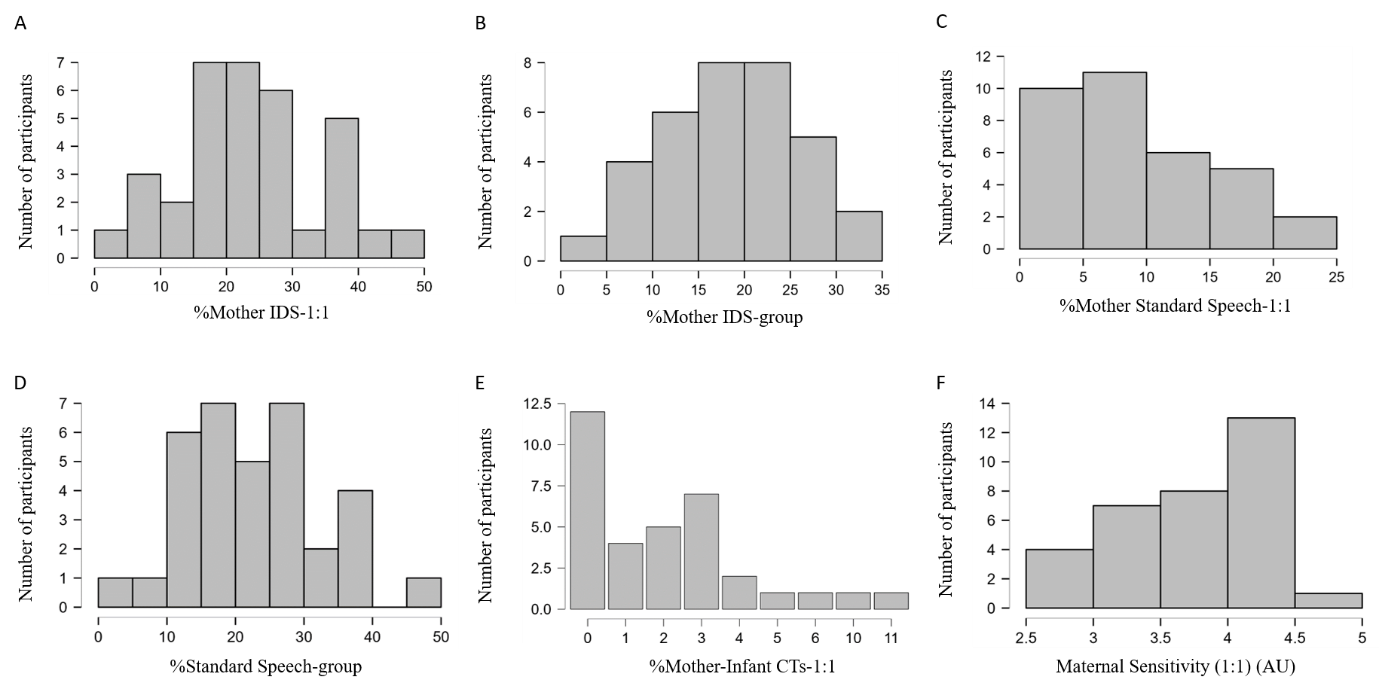
**

**Figure S1. Distribution of participants’ speech input measurements and maternal sensitivity at 3 months of age.** (A) %Mother Infant directed speech (IDS) during one-on-one interaction (B) %Mother Infant directed speech (IDS) in a group context (C) %Mother Standard Speech during one-on-one interaction (D) %Standard Speech in a group and (E) %Conversational turn (CTs) during one-on-one mother-infant interaction; (F) Maternal Sensitivity during mother-infant face-to-face free interaction in the lab.

**Table S1.**

*Correlations between social interaction variables (LENA and CIB) assessed at 3 months of age and Vocabulary Raw scores*

| Social interaction variable | Vocabulary  18 M | Vocabulary  21 M | Vocabulary  24 M | Vocabulary  27 M | Vocabulary  30 M |
| --- | --- | --- | --- | --- | --- |
| %Mother IDS-1:1 | *r* = 0.25  *p* = 0.15 | *r* = 0.21  *p* = 0.23 | *r* = 0.42  *p* = 0.014* | *r* = 0.45  *p* = 0.008** | *r* = 0.43  *p* = 0.012* |
| %Mother IDS-group | *r* = -0.03  *p* = 0.88 | *r* = - 017  *p* = 0.33 | *r* = -0.04  *p* = 0.82 | *r* = 0.00  *p* = 0.99 | *r* = 0.06  *p* = 0.75 |
| %Mother Standard Speech-1:1 | *r* = 0.06  *p* = 0.73 | *r* = 0.09  *p* = 0.61 | *r* = 0.18  *p* = 0.31 | *r* = 0.17  *p* = 0.33 | *r* = 0.12  *p* = 0.50 |
| %Standard Speech- group | *r* = -0.14  *p* = 0.42 | *r* = -0.29  *p* = 0.09+ | *r* = -0.28  *p* = 0.10 | *r* = -0.31  *p* = 0.08+ | *r* = -0.29  *p* = 0. 09+ |
| %Mother-Infant CTs-1:1 | *r* = 0.26  *p* = 0.14 | *r* = 0.19  *p* = 0.28 | *r* = 0.27  *p* = 0.12 | *r* = 0.35  *p* = 0.04 * | *r* = 0.35  *p* = 0.04 * |
| Maternal Sensitivity (CIB) | *r* = 0.39  *p* = 0.024* | *r* = 0.31  *p* = 0.08+ | *r* = 0.41  *p* = 0.017* | *r* = 0.47  *p* = 0.005 ** | *r* = 0.43  *p* = 0.013* |

Note: +p <0.1, * p < .05, ** p < .01

**Table S2**

*Correlations between social interaction variables (LENA and CIB) assessed at 3 months of age and Vocabulary percentile scores, controlling for SES*

| Variable | Vocabulary  18 M | Vocabulary  21 M | Vocabulary  24 M | Vocabulary  27 M | Vocabulary  30 M |
| --- | --- | --- | --- | --- | --- |
| %Mother IDS-1:1 | *r* = 0.31,  *p* = 0.08 | *r* = 0.33,  *p* = 0.065 | *r* = 0.47,  *p* = 0.006** | *r* = 0.53,  *p* = 0.002** | *r* = 0.51,  *p* = 0.003** |
| %Mother IDS-group | *r* = -0.05  *p* = 0.79 | *r* = -0.17  *p* = 0.34 | *r* = -0.08  *p* = 0.66 | *r* = -0.03  *p* = 0.87 | *r* = 0.004  *p* = 0.98 |
| %Mother Standard Speech-1:1 | *r* = 0.08,  *p* = 0.66 | *r* = 0.21,  *p* = 0.25 | *r* = 0.17,  *p* = 0.34 | *r* = 0.16,  *p* = 0.36 | *r* = 0.10,  *p* = 0.58 |
| %Standard Speech- group | *r* = -0.18,  *p* = 0.33 | *r* = -0.33,  *p* = 0.06 | *r* = -0.36,  *p* = 0.038* | *r* = -0.39,  *p* = 0.026* | *r* = -0.39,  *p* = 0.024* |
| %Mother-Infant CTs-1:1 | *r* = 0.26,  *p* = 0.14 | *r* = 0.21,  *p* = 0.24 | *r* = 0.31,  *p* = 0.08 | *r* = 0.38,  *p* = 0.03* | *r* = 0.41,  *p* = 0.017* |
| Maternal Sensitivity (CIB) | *r* = 0.43,  *p* = 0.014* | *r* = 0.36,  *p* = 0.045* | *r* = 0.44,  *p* = 0.013* | *r* = 0.5,  *p* = 0.003** | *r* = 0.45,  *p* = 0.01* |

*Note: * p < .05, ** p < .01*

**Table S3.**

*Correlations between social interaction variables (LENA and CIB) assessed at 3 months of age and Irregular Words percentile scores*

| Variable | Irregular  Words  18 M | Irregular Words  21 M | Irregular Words  24 M | Irregular Words  27 M | Irregular Words  30 M |
| --- | --- | --- | --- | --- | --- |
| %Mother IDS-1:1 | *r* = 0.19,  *p* = 0.29 | *r* = 0.20,  *p* = 0.25 | *r* = 0.46,  *p* = 0.006** | *r* = 0.53,  *p* = 0.001** | *r* = 0.65,  *p* > 0.001 *** |
| %Mother IDS-group | *r* = -0.1  *p* = 0.57 | *r* = -0.08  *p* = 0.64 | *r* = -0.02  *p* = 0.92 | *r* = 0.05  *p* = 0.76 | *r* = 0.1  *p* = 0.53 |
| %Mother Standard Speech-1:1 | *r* = 0.09,  *p* = 0.62 | *r* = 0.14,  *p* = 0.43 | *r* = 0.22,  *p* = 0.21 | *r* = 0.28,  *p* = 0.11 | *r* = 0.29,  *p* = 0.10 |
| %Standard Speech- group | *r* = -0.15,  *p* = 0.40 | *r* = -0.18,  *p* = 0.31 | *r* = -0.29,  *p* = 0.10 | *r* = -0.19,  *p* = 0.29 | *r* = -0.26,  *p* = 0.14 |
| %Mother-Infant CTs-1:1 | *r* = 0.23,  *p* = 0.18 | *r* = 0.33,  *p* = 0.06 | *r* = 0.34,  *p* = 0.053 | *r* = 0.46,  *p* = 0.006** | *r* = 0.43,  *p* = 0.011* |
| Maternal Sensitivity (CIB) | *r* = 0.40,  *p* = 0.02* | *r* = 0.44,  *p* = 0.01* | *r* = 0.49,  *p* = 0.003* | *r* = 0.56,  *p* < 0.001 *** | *r* = 0.48,  *p* =0.005** |

*Note: * p < .05, ** p < .01, *** p < 0.001*

**Supplemental Statistical Analyses**

We conducted Spearman correlations between speech input measures and Maternal Sensitivity and Irregular Words percentile scores (Table S3). The analysis revealed that %Mother IDS-1:1 at 3 months of age is significantly associated with irregular words scores at 24, 27 and 30 months of age (*N* = 34, all *r*_s_ > 0.45, all *p*s < 0.007) but not with language scores before 24 months of age. %Mother IDS-group was not associated with irregular words percentile scores (N=34, all |*r*_s_| < 0.1, all *p*s > 0.5). %Mother Standard speech-1:1 was not associated with irregular words percentile scores (N=34, all *r*_s_ < 0.3, all *p*s > 0.1). %Standard Speech-group was not associated with irregular words percentile scores (N=34, all |*r*_s_| < 0.3, all *p*s > 0.09).

%Mother-Infant CTs-1:1 at 3 months of age is positively associated with irregular words percentile in 27-months-olds (N = 34, *r*_s_ = 0.46, *p* = 0.006) and in 30-months-olds (N = 34, *r* = 0.43, *p* = 0.011). Maternal Sensitivity at 3 months of age is associated with Irregular Words percentile scores through all measured language development stages, from ages 18 to 30 months of age (*N* = 33, all *r*_s_ > 0.4, all *p*s < 0.02), where the strongest correlation was with 27 months of age (*r*_s_ = 0.56, *p* < 0.001). Because the two speech input variables, %Mother IDS-1:1 and %Mother-Infant CTs-1:1, are significantly inter-correlated (*r*_s_ = 0.45, *p* = 0.007), we further explored the obtained correlations accounting the effect of the other variable using partial Spearman correlation. The analysis revealed no significant contribution for %Mother-Infant CTs-1:1beyond %Mother IDS-1:1 in the association with Irregular Words percentile scores at all measured time points (%Mother-Infant CTs-1:1 conditioned on %Mother IDS-1:1, all *r*_s_ < 0.3, all *p*s > 0.1). However, a partial Spearman correlation revealed a significant contribution for %Mother IDS-1:1 in association with Irregular Words percentile scores at 24, 27 and 30 months of age (%Mother IDS-1:1 conditioned on %Mother-Infant CTs-1:1, all three-time points *r*_s_ > 0.37, all *p*s < 0.035).

Since Maternal Sensitivity and %Mother-Infant CTs-1:1are significantly inter-correlated (*r* = 0.43, *p* = 0.013), we further explored the obtained correlations accounting the effect of the other variable using partial Spearman correlation. The analysis revealed no significant contribution for %CTs beyond Maternal Sensitivity in the association with Irregular Words percentile scores in all measured time points (%Mother-Infant CTs-1:1 conditioned on sensitivity, all *r*_s_ < 0.25, all *p*s > 0.2). However, a partial Spearman correlation revealed a significant contribution for Maternal Sensitivity in association with Irregular Words percentile scores in all measured time points (Maternal Sensitivity conditioned on %Mother-Infant CTs-1:1, all *r*_s_ > 0.36, all *p*s < 0.042).
